# Microfluidic Protein Imaging Platform: Study of Tau Protein Aggregation and Alzheimer’s Drug Response

**DOI:** 10.3390/bioengineering7040162

**Published:** 2020-12-13

**Authors:** Shubha Jain, Sarpras Swain, Lopamudra Das, Sarita Swain, Lopamudra Giri, Anand Kumar Kondapi, Harikrishnan Narayanan Unni

**Affiliations:** 1 Department of Biomedical Engineering, Indian Institute of Technology Hyderabad, Hyderabad 502285, India; bm14resch11005@iith.ac.in (S.J.); lopamudra2806@gmail.com (L.D.); 2 Department of Chemical Engineering, Indian Institute of Technology Hyderabad, Hyderabad 502285, India; ch14resch01002@iith.ac.in (S.S.); giril@che.iith.ac.in (L.G.); 3 Department of Biotechnology and Bioinformatics, University of Hyderabad, Hyderabad 500046, India; swain.sarita27@gmail.com (S.S.); akondapi@uohyd.ac.in (A.K.K.)

**Keywords:** tau protein, Alzheimer’s disease, microfluidics

## Abstract

Tau protein aggregation is identified as one of the key phenomena associated with the onset and progression of Alzheimer’s disease. In the present study, we performed on-chip confocal imaging of tau protein aggregation and tau–drug interactions using a spiral-shaped passive micromixing platform. Numerical simulations and experiments were performed in order to validate the performance of the micromixer design. We performed molecular modeling of adenosine triphosphate (ATP)-induced tau aggregation in order to successfully validate the concept of helical tau filament formation. Tau aggregation and native tau restoration were realized using an immunofluorescence antibody assay. The dose–response behavior of an Alzheimer’s drug, methylthioninium chloride (MTC), was monitored on-chip for defining the optimum concentration of the drug. The proposed device was tested for reliability and repeatability of on-chip tau imaging. The amount of the tau protein sample used in our experiments was significantly less than the usage for conventional techniques, and the whole protein–drug assay was realized in less than two hours. We identified that intensity-based tau imaging could be used to study Alzheimer’s drug response. In addition, it was demonstrated that cell-free, microfluidic tau protein assays could be used as potential on-chip drug evaluation tools for Alzheimer’s disease.

## 1. Introduction

Alzheimer’s disease (AD) is one of the most common fatal neurological disorders affecting the lives of millions worldwide [1,2,3]. The development of an effective treatment for AD is still in the budding phase and is still limited by an incomplete understanding of Alzheimer’s biology [4,5]. A significant amount of recent research is focused on the role of tau protein in neurodegeneration, in effort to develop viable treatments for various neuropathological conditions [6,7]. In vitro study of AD requires the development of animal models and biomimicking models that can represent the biomolecular pathways associated with the deterioration of neurons. One of the key features of this neurodegenerative disease is the accumulation of defective tau protein in the neuronal space [8]. 

The normal biological activity of tau is suppressed by hyperphosphorylation. In the case of Alzheimer’s patients, tau protein is approximately three- to four-fold more hyperphosphorylated and aggregated into bundles of filaments than the native tau found in adult brains [8]. Tau pathology involves the formation of neuronal tangles of paired helical filaments (PHFs) and straight filaments partly constituted of hyperphosphorylated tau protein [9]. The hyperphosphorylated protein interferes with the native function of tau and ultimately cause cells to undergo apoptosis. The N-terminal region of tau protein has a putative site for adenosine triphosphate (ATP) binding, and this passive phosphorylation of tau by ATP induces tau self-assembly into AD filaments [10]. In this regard, research focused on the identification of aggregated tau as a useful biomarker [11] has paved the way to novel therapeutic strategies for Alzheimer’s disease based on the inhibition of tau aggregation [12].

Numerous drugs target the inhibition of tau aggregation. Methylthioninium chloride (MTC) or methylene blue (MB) is used for the treatment of medical conditions such as septic shock, methemoglobinemia, and plasmodium infection. MTC has emerged as a promising candidate as a tau aggregation inhibitor. Recent evidence suggests MTC as a tau aggregation inhibitor in both in vitro as well as in vivo cellular models and transgenic mice [12]. 

Chip-scale in vitro models aimed at understanding protein dynamics are immensely useful in the development of drug screening platforms. Microfluidic approaches for studying protein folding and aggregation have been reported in the literature only recently [13]. Li et al. [14] developed an ultrafast continuous flow-based micromixer to track the early folding kinetics of G-quadruplex. The micromixer was developed to investigate the hairpin formation in the early folding process of the human telomere G-quadruplex. A T-shaped micromixer with consecutive w-shaped structures was developed by Li et al. [15] to study macromolecule kinetics in molecular crowding conditions present in high viscosity fluids. Perry et al. [16] reported a microfluidic approach for the determination of protein structure via crystallization screening and optimization. Waldauer et al. [17] developed a rapid microfluidic mixer to study protein folding. The flow-focusing microfluidic geometry caused rapid constriction of the protein flow into a narrow jet with a width of around 100 nm. The difference in diffusion constants of protein and denaturant was responsible for the rapid dilution of the denaturant from the focused protein stream. Microsecond protein folding events revealed by time-resolved fluorescence resonance energy transfer were studied using a microfluidic mixer developed by Jiang et al. [18]. A three-dimensional micromixer for studying the kinetics of macromolecule folding under molecular crowding conditions was developed by Liu et al. [19]. The fabricated device employed a U-shaped channel design to generate microvortices inside the channels. Additionally, there have been several microfluidic platforms available that used fluorescence and laser scanning microscopes for the detection and quantification of protein from whole blood [20,21]. However, the investigation of protein aggregation and drug testing on-chip is rather limited. 

The development of in vitro models based on neuron culture and differentiation is cumbersome and complicated; hence, there is an increasing demand for developing cell-free assays for studying drug–protein interactions [22,23,24]. On the other hand, the cost of neuroprotein being very high and the reproducibility studies in conventional vials remain challenging. Moreover, protein analysis using conventional methods such as NMR and circular dichroism requires high sample volumes, and it is hard to achieve such a volume in a protein-based study. Protein studies using laser scanning confocal microscopy (LSCM) require samples in the range 40–50 µL in general. However, due to the small sample volume on a coverslip the protein solution is dried faster, making reaction inconvenient. Chip-scale cell-free assays for protein studies are quite useful in this scenario, as they can be implemented as cost-effective platforms for drug testing. 

In the present study, we aim to investigate tau aggregation and dose–response of MTC using a chip-scale micromixing device. We monitored tau aggregation and restoration on-chip, where the extent of protein aggregation was correlated with the change in fluorescence intensity. Molecular modeling was performed to demonstrate tau protein structural changes (filament formation) resulting from ATP binding. Subsequently, the tau–MTC interaction was studied on-chip, and drug dose optimization was performed. A continuous flow-based passive microfluidic mixer was designed and fabricated for this purpose. The mixing device has a spiral-shaped geometry that helps achieve sample mixing due to Dean secondary vortex flows [25]. The longest human tau isoform containing 441 (human, recombinant) residues [26] was chosen for performing experiments, because it has a high proportion of phosphorylable serine and threonine residues. To the best of our understanding, reports on microfluidic cell-free assays for tau protein aggregation and evaluating Alzheimer’s drug responses are minimal in the literature. Miniaturized platforms for protein aggregation can be utilized as cost-effective cell-free assays for drug screening; this argument forms the basis of the present work.

## 2. Materials and Methods

### 2.1. Schematic of Microfluidic Assay for Tau Aggregation and Drug Response

**Figure 1:**
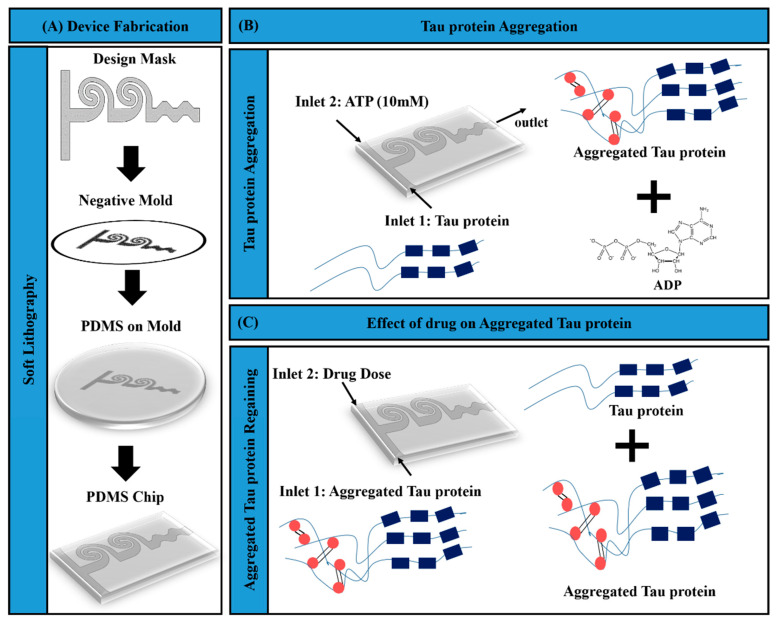
Schematic representing the micromixer device fabrication and experimental setup. (**A**) Soft lithography process. (**B**) Adenosine triphosphate (ATP)-induced tau aggregation performed on-chip. (**C**) Tau–drug experiments and tau recovery performed using the fabricated chip. The blue rectangles represent the peptide repeats in native tau protein and red circles represent the entangled area of the aggregated tau protein.

The microfluidic device was designed based on the principle of passive microfluidic mixing and the device has spiral-shaped geometry. The schematic of the proposed microfluidic protein imaging assay and related fabrication is depicted in Figure 1. Soft lithography fabrication is outlined in Figure 1A, while processes of tau–ATP aggregation and tau–MTC interaction realized in the microfluidic mixing device are presented in Figure 1B. The schematic of the antibody assay and the imaging area (closer to the device outlet) is presented in Figure 1C.

### 2.2. Fabrication of Microfluidic Mixer

The height of the fabricated microfluidic channel was 50 µm. Standard soft lithography was employed for the fabrication of the device (Figure 1A). Cleaned and dehydrated silicon wafer was spin coated with negative photoresist SU-8 with a thickness of 50 µm. SU-8 coated wafer was soft-baked at 65 °C for 3 min and 95 °C for 6 min. Further, the processed wafer was exposed inside a maskless Laser writer (Dilase-250) to produce the mold. Subsequently, a post-bake treatment was performed at 65 °C for 2 min and 95 °C for 6 min. The master mold was obtained after the development process. Once the mold was prepared, the PDMS (Polydimethylsiloxane) prepolymer base (Sylgard 184 from Dow Corning) and curing agent with the proportion of 10:1 ratio was poured onto the SU-8 master mold. The mold was degassed and cured at 70 °C. Subsequently, the PDMS replica obtained from the SU-8 master mold was plasma-bonded to precleaned glass substrate. The size of the fabricated microfluidic mixer is demonstrated in supplementary Figure S1.

### 2.3. Modeling and Validation of Microfluidic Mixer Design

The efficiency of biological mixing is vital to the performance of protein–drug assays. Micromixer dimensions were optimized by performing a Finite element-based numerical simulation. The micromixer geometry was discretized into 13,283 triangular mesh elements. Coupled field equations of fluid flow and mass transport were solved using COMSOL Multiphysics in order to simulate the concentration field inside the microchannels. For simulations, the flow velocity at the channel inlet was considered in accordance with the value maintained during microfluidic assay experiments. The performance of the spiral microfluidic design was validated by performing experiments using colored dye solutions. Fluid flow rates were maintained at 20 µL per hour using a multifeed syringe pump (Cole Parmer, Vernon Hills, IL, USA). Digital images were captured using a high-resolution camera (DSLR). The images of various cross-sections (Regions of interest—ROIs) along the channel length were captured at identical ambient illumination conditions. The efficiency of micromixing was determined by the amount of resultant color gradient generated. The captured images were processed using the software ImageJ to measure their intensity (total color, red, blue, green, and grayscale intensity). The percentage of mixing was calculated by the equation:

(1) $$\%\;\mathrm{of}\;\mathrm{mixing}=\left(1-\;\left(\frac{\mathrm{Intensity}\;\mathrm{at}\;\mathrm{inlet}-\mathrm{Intensity}\;\mathrm{at}\;\mathrm{ROI}}{\mathrm{Intensity}\;\mathrm{at}\;\mathrm{Inlet}}\right)\right)\;\times 100$$

### 2.4. Molecular Modeling of Tau Protein Aggregation

We performed molecular simulations to validate the concept of ATP-induced aggregation of tau which is employed in the experiments involving microfluidic antibody assays. The structure data format (SDF) 3D structure of ATP was retrieved from the NCBI PubChem database along with the corresponding PubChem ID, molecular weight, and molecular formula. The ATP was converted into PDB (Protein data bank) format using the PyMol tool, Discovery Studio v4.1 tools, and online SMILES translator web server as per requirement. The tau protein structure was retrieved from the protein data bank. 

### 2.5. Microfluidic Experiments for Tau Imaging

Tau imaging experiments were performed using the fabricated microfluidic design. The prime reason for choosing ATP to induce tau aggregation was based on the nature of tau–ATP interactions and its relevance to Alzheimer’s. It is reported that a tau interaction with a 10 mM concentration of ATP results in the formation of helical tau filaments [10], while concentrations much lower than ATP (1 mM) do not correspond to lateral interactions, bundling, and twisting of tau filaments. We used MES buffer (2-(N-morpholino)ethanesulfonic acid; Sigma) for the dilution of ATP and DI water (Deionized water) was used for making the tau protein solution. Tau protein (Sigma) and 10 mM ATP (Sigma) samples were introduced through the device inlets. Tau imaging was performed in the imaging chamber close to the channel outlet. Subsequently, the aggregated tau samples from the above-mentioned experiment were utilized for performing tau–drug experiments (as indicated in Figure 1). The experiments were performed using a multiple syringe pump, where a constant flow rate of 20 µL per hour was maintained. The sample incubation period required in our experiments was 1 h. The microfluidic inlet flow rates were maintained such that the mixed samples reached the imaging outlet in 1 h. The primary intention of the tau–drug experiment was to image tau protein aggregation and restoration on-chip in order to optimize the MTC dosage. Aggregated tau and MTC were introduced through the device inlets to perform sample mixing. Subsequently, tau images corresponding to various dosages of MTC were captured close to the channel outlet (imaging window). 

**Figure 2:**
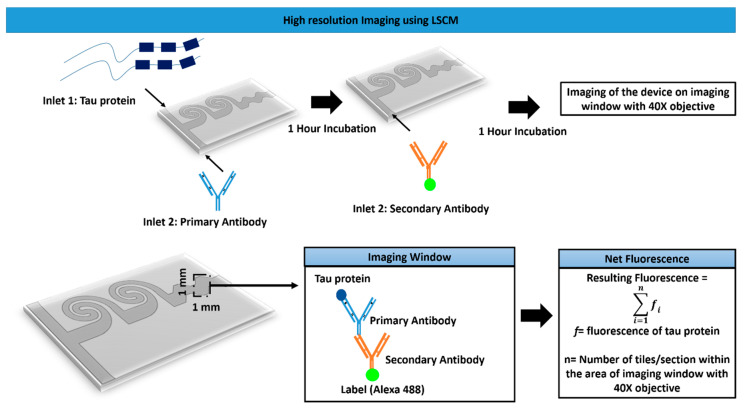
Schematics of protein imaging using laser scanning and fluorescence quantification inside the fabricated chip.

The schematic of the imaging assay is depicted in Figure 2. The details of the imaging window closer to the device outlet are presented. The immunofluorescence assay was performed using the primary antibody for tau protein. The microfluidic channel was treated with plasma to ensure the adhesion of tau protein while performing the immunoassay. Initially, the tau protein sample was incubated for 45 min with a primary anti-tau antibody (Sigma). Subsequently, a secondary antibody conjugated with Alexa 488 (Thermo Fisher Scientific, Waltham, MA, USA) was introduced after washing the primary antibody. The resulting sample was incubated for another 45 min. The non-attached secondary antibody was removed by flushing the channels using PBS buffer before the images were captured. Tau imaging was performed using a laser scanning confocal microscope (LSCM—Leica TCS SP8, Wetzlar, Germany) with an argon laser at 488 nm excitation, and emission was recorded at 510 nm. Images were captured using a 40× objective, and the imaging location closer to the outlet of the microfluidic channel is referred to as “imaging window”. The intensity of fluorescence regaining corresponding to various drug dosages was measured. In the above experiments, we used non-aggregated tau protein as control, using the above-mentioned immunofluorescence method to visualize the sample under the microscope.

The net resulting fluorescence was calculated from various sections under the imaging window of 1 mm × 1 mm using the formula

(2) $$Net\;Fluorescence=\overset{n}{\underset{i=1}{\sum }}f_{i}$$

where,

*f* = fluorescence of tau protein;
*n* = number of tiles/sections within the area of the imaging window.

The data acquisition was performed using Leica LAS X software. The tile-scanning within the imaging window and evaluation of the whole intensity within these regions was carried out to ensure consistency in the results. 

Optimization of the drug concentration was performed by one factorial design using design expert software (DOE; Stat-Ease Inc., Minneapolis, MN, USA). An optimal and statistical interpretation of results with minimum experiments is possible using DOE, and hence the cost of experiments can be reduced with efficient use of the protein. The extent of tau protein regeneration was evaluated by considering the drug dose as an input factor, and percentage change in fluorescence intensity was used as a response (DOE; Table 1). The selection criteria were to achieve maximum fluorescence intensity corresponding to the minimum drug concentration. A tau protein concentration of 1 µg/µL was utilized for performing the entire range of experiments involving different dosages of MTC. It can be noted that the amount of tau protein requirement for a single experiment is much less than 1µg, making the current microfluidic design a useful alternative as a cost-effective drug testing platform. Larger amounts of tau samples are normally required (in the range of milligrams) in the case of non-microfluidic in vitro experiments [27].

## 3. Results and Discussion

### 3.1. Micromixing Simulations

**Figure 3:**
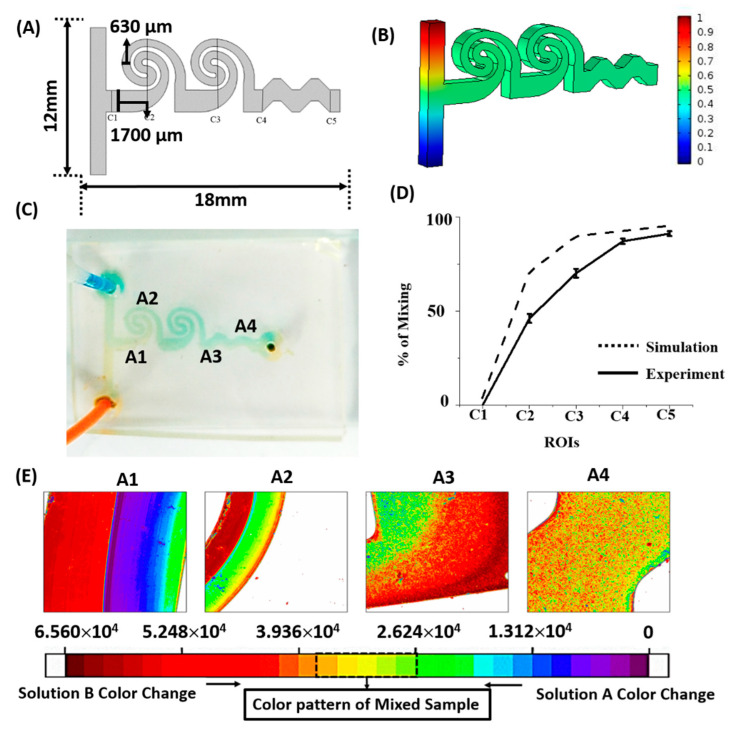
(**A**) Schematic of the spiral microfluidic channel and regions of interest (ROIs). (**B**) Microfluidic mixing simulation results—the colour bar indicates the concentration field inside the microfluidic channels. The resultant concentration value close to 0.5 represents the optimum result for the adequate mixing of samples. (**C**) Experimental result of dye mixing in spiral microfluidic design. (**D**) Percentage of mixing compared between simulation and experiments. (**E**) Spatial intensity map of various regions in the spiral channel, illustrating the mixing pattern.

The results of micromixing simulations and microfluidic dye mixing experiments are presented in Figure 3. Sample concentrations at various locations along the length of the channel (Figure 3A) are presented. Efficient mixing in areas closer to assay imaging can be observed from the simulation results (Figure 3B) and is validated by the mixing experiments performed using dye solutions (Figure 3C). We identified regions of interest (ROIs) along the length of the channel (C1–C5), and the percentage of mixing corresponding to these regions was calculated using Equation (1). The comparison between simulated and experimental concentration values is presented in Figure 3D, which noticeably indicates reasonably good mixing in areas (C4–C5) close to the channel outlet where tau imaging was performed. The spatial intensity map (Figure 3E) was plotted for the visualization of the mixing pattern in the channel, as presented in Figure 3C. The color pattern close to the channel outlet (section A4) also indicates efficient mixing in the spiral microfluidic design. However, from Figure 3D, it can be observed that the computed mixing efficiency is higher than the experimental values, specifically in areas represented by the spiral sections (C2–C4). One reason for this deviation can be attributed to flow development effects, while fully developed flow conditions were assumed when performing the numerical simulations. In addition, syringe pump lag may also have contributed to marginally differing flow rates of two fluids in the channel, affecting the mixing performance. In numerical simulations, a uniform flow rate at both inlets was assumed. Another relevant factor could be the assumption of perfectly vertical walls and square cross-sections of microfluidic channels, while the fabricated device can have cross-sections deviating marginally from a perfect square. 

### 3.2. Tau Aggregation—Molecular Simulation and Experiments

**Figure 4:**
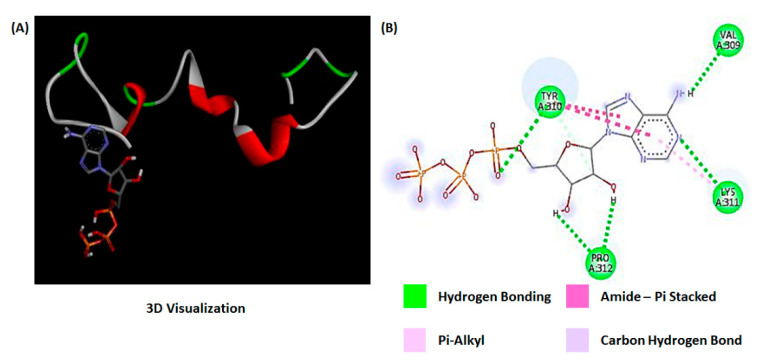
Protein–ATP interaction using molecular docking simulation. (**A**) 3D interaction between molecules visualized using discovery studio visualizer. (**B**) Interaction and bonding between amino acids in the aggregate structure.

The process of tau aggregation and the resultant conformation was simulated using the molecular docking method present in the bioinformatics toolbox. Docking was performed for the binding of ATP and tau protein. The docking simulation results revealed important parameters related to binding interactions such as binding energy, ligand efficiency, inhibition constant, and electrostatic energy for four ligands/drug potential targets interactions. The values of these parameters are presented in Table 2. The molecular docking studies indicated that the drug–target interactions of tau–ATP were weakly bonded (binding energy: −0.68 kcal/mol) by hydrogen bond, and the interaction site of the hydrogen bond involves Val309, Tyr310, Lys311, and Pro312 (Figure 4B). The resulting molecular structure from tau–ATP interactions was visualized using discovery studio tools (Figure 4A). The modeling results indicate tau aggregation pattern and twisted filament formation, which is identical to the aggregation pattern found in Alzheimer’s disease. 

**Figure 5:**
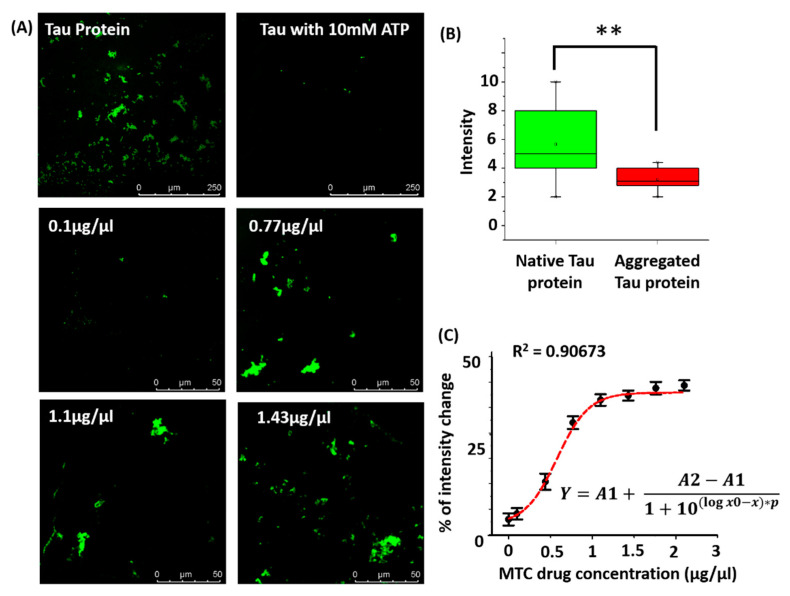
(**A**) Confocal images of native tau, aggregated tau and methylthioninium chloride (MTC) drug response. (**B**) Box plot of intensity corresponding to native and aggregated tau protein. The reduction in image intensity of tau resulting from ATP binding is observed in Figure 5B. Data were analyzed using *t*-test, ** *p* < 0.005. (**C**) MTC drug response which indicates the percentage change in fluorescence intensity corresponding to different concentrations of the drug. Data are fitted with a polynomial equation.

Images of native tau and aggregated tau (Figure 5A, two images on top) were captured using LSCM, and the box plot (Figure 5B) represents the corresponding change in fluorescence intensity (normalized). The reduction in fluorescence intensity resulting from ATP-induced aggregation can be observed from the confocal images as well as from the intensity box plot. The intensity of aggregated tau protein is reduced several folds because of ATP binding, as observed from Figure 4B. The molecular simulation results and the tau–ATP microfluidic imaging experiments provide complementing information regarding tau aggregation. 

### 3.3. Drug Dose Optimization 

In this section, we describe the results of on-chip tau imaging and optimization of MTC response. Figure 5A presents the confocal images of tau protein, where the first two images represent native tau and ATP-induced aggregated tau. The images below in Figure 5A highlight the MTC response on aggregated tau. Figure 5C depicts the dose–response behavior of MTC. The changes in image intensity in response to MTC dosages in the range of 0.1–2.1 µg/µL are presented. Tau restoration is observed to be minimal corresponding to lower dosages of the drug. We have observed that the intensity patterns of native tau and regained tau (corresponding to drug dose of 1.43 µg/µL) are identical (details are presented in supplementary Figure S2). In the clinical scenario, the major requirement would be to use the minimum dosage of a drug that corresponds to the maximum efficacy. In our experiments, the recovery of native tau is represented by a change in fluorescence image intensity. Therefore, we propose that the optimum amount of the drug can be determined by calculating the minimum dosage of the drug corresponding to the maximum percentage of change in intensity.

**Figure 6:**
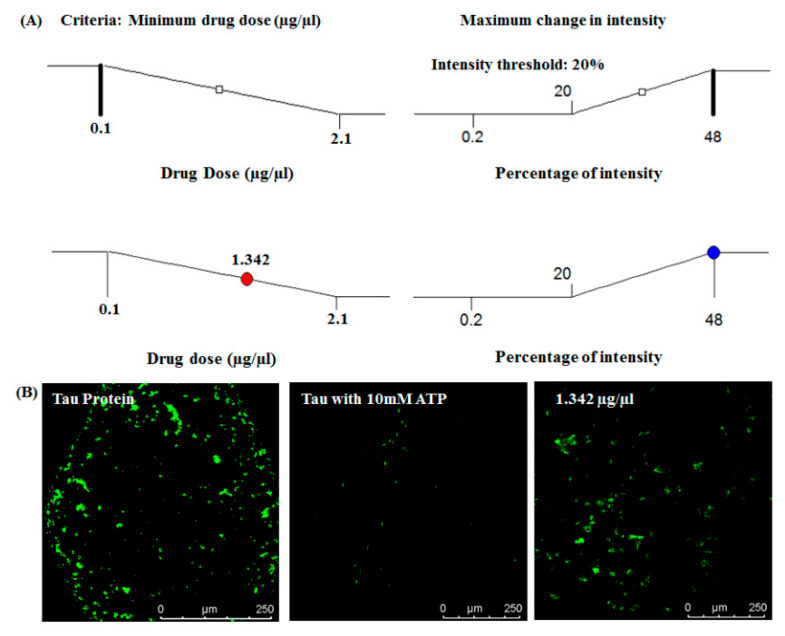
Optimized drug dose concentration. (**A**) Ramp plot of drug dose optimization corresponding to minimum drug dosage obtaining maximum intensity (lowest allowed intensity is 20%). (**B**) Confocal imaging at optimized drug dose—the optimized drug dose used was 1.342 µg/µL. Tau protein without ATP treatment is considered as the positive control, and tau with 10 mM ATP is considered as the negative control.

The Ramp plot (Figure 6A) presents the range of intensity change in response to the change in drug dosage. No significant change in image intensity was observed below the intensity value of 20%. Hence, we identified an acceptable minimum intensity change at 20%. This observation indicates that any value of MTC higher than the value corresponding to a 20% intensity will result in a nearly equal level of tau restoration. Hence, the optimum dosage of the drug is calculated as 1.342 µg/µL. MTC dosages lower than the optimum value indicate a low fluorescence intensity, meaning a larger amount of aggregated tau protein. Figure 6B depicts the image corresponding to the optimized dose of MTC, in addition to images of control. It should be pointed out that the dosage–intensity relation might differ in the case of another Alzheimer’s drug, because the tau–drug interaction pattern will differ, and the corresponding threshold intensity may as well change. 

**Figure 7:**
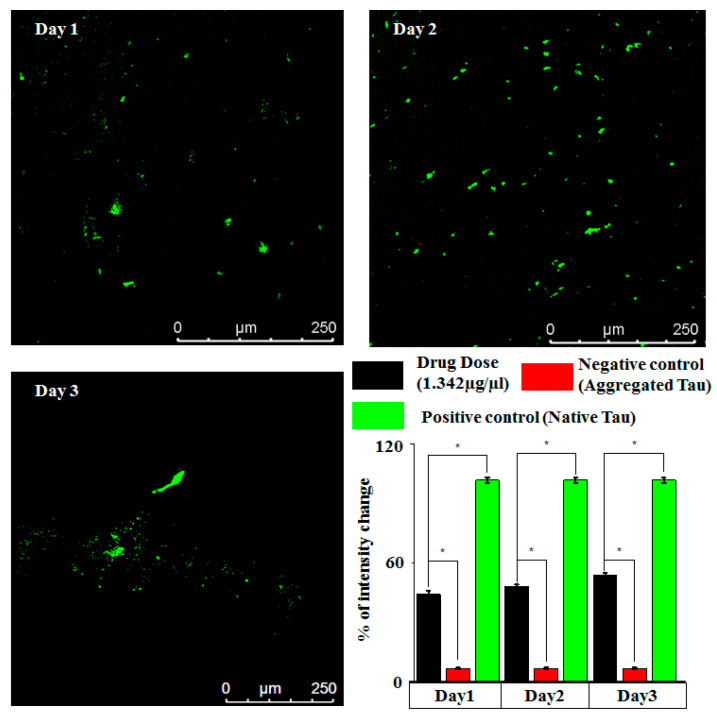
Confocal images captured on a day to day basis (using optimized MTC drug dose) to ascertain the reproducibility of tau–MTC experimental results achieved on-chip. Data were analyzed using *t*-test, * *p* < 0.05.

Consistency of results is particularly significant if the designed microfluidic assay is argued as an on-chip platform for drug testing. In order to test the reproducibility of the tau imaging results, we performed tau–drug experiments on-chip using an optimized drug dose concentration (1.342 µg/µL) on a day to day basis. The confocal images were taken from three different chips on consecutive days, where the imaging location within the imaging window remained the same. The results are presented in Figure 7. It is observed that the results are reasonably reproducible, as no significant variation in intensity could be observed among these images. The bar plot in Figure 7 indicates that there is no significant change in the drug response (represented by fluorescence intensity) as is indicated from day to day variability experiments. This result indicates that the proposed microfluidic platform is suitable for performing tau–drug experiments with repeatability, and could be suitable for testing Alzheimer’s drugs.

### 3.4. On-Chip Tau Aggregation and Neurological Implications

As identified from Figure 5A, the intensity-based images of native tau protein (control) and aggregated tau protein indicate a distinct pattern. The intensity of native tau is higher due to the binding of the anti-tau antibody and conjugated Alexa 488. However, ATP conjugation alters the pattern of antibody binding to tau protein. The binding of ATP to tau results in helical filament formation, altering the local structure of tau. This causes the hindrance in the binding of primary and secondary antibody to tau protein. This effect is highlighted by a reduction in fluorescence intensity of aggregated tau, as observed in Figure 5A (images on top). The MTC drug interferes with the mutual binding of tau necessary for aggregation and helical filament formation. The aggregated tau is reversed into native tau upon interaction with MTC, resulting in proper binding of anti-tau antibody (capture antibody) and secondary antibody. The neuronal microtubule environment contains tubulin, and hence the role of this protein on the dynamics of tau can present an interesting question in this regard. Ideally, tau protein interacts with tubulin—the microtubule protein present in the neuronal microenvironment. However, it is reported that MTC reverses the proteolytic stability of tau filaments isolated from AD brain tissues in vitro, without disrupting normal tau–tubulin interactions [28]. This essentially means that the MTC–tau interaction and recovery of native tau is virtually independent of tau–tubulin interactions. Therefore, MTC drug response data from the proposed in vitro model could be biologically significant, even when tau–tubulin interaction is not considered in the present model. Amyloid-β protein outside the neuronal space is yet another hallmark of AD progression [29]. However, a biologically realistic study of Amyloid-β should involve the culture of microglial cells, as they are phagocytotic to Amyloid-β deposits [30]. This would in turn limit the possibility of developing a cell-free microfluidic assay. Therefore, targeting tau aggregation likely remains the feasible option for cost-effective on-chip drug assays for AD. The recent failure of drugs aimed at controlling Amyloid-β accumulation also fortifies this observation [31]. The proposed chip-scale platform for studying tau–drug responses could prove beneficial in experimental modeling of Alzheimer’s disease. Using the methodology outlined in the present work, biological fluids containing tau protein can be utilized for performing experiments to understand the detailed dynamics of tau in Alzheimer’s. As a cell-free assay, one of the limitations of the present device is that spread of tau through communicating neurons cannot be represented. However, the proposed microfluidic design can also be upgraded for on-chip neuronal culture [32] for studying the role of tau protein in axonal degeneration [33] using a cell-based assay. Moreover, the drugs selected through the cell-free assay can be further confirmed for application toward axonal regeneration. Another limitation of the current work is that it does not focus on the analysis of the protein aggregate size for three different conditions—i.e., normal tau protein, aggregated tau protein in presence of ATP, and regained tau protein in presence of MTC. In future, the size analysis can be performed to obtain a detailed insight on the role of aggregate size on tau protein dynamics and extent of native tau recovery. Additionally, the present work can be extended for the screening of multiple Alzheimer’s drugs with suitable modification of the proposed design, where the performance of multiple drugs can be evaluated simultaneously. In the present work, we proposed a quantification scheme for estimating tau protein concentration using average fluorescent intensity from images present in a larger section (details are presented in supplementary Figure S3). In the future, machine learning algorithms can be implemented to account for factors such as non-uniformity in tau intensity and tau aggregate size present in the images, so that a better calibration curve can be obtained.

## 4. Conclusions

Protein aggregation experiments using conventional methods can be expensive, time-consuming, and require a high amount of protein to perform various experiments. Although tau aggregation is of paramount importance in understanding the dynamics of Alzheimer’s, the construction of cell-based models needs an expensive set up for performing in vitro experiments on neurodegeneration and drug response. In this context, we proposed a cost-effective microfluidic antibody assay to study ATP-induced tau protein aggregation and MTC drug response. A spiral microfluidic mixer-based antibody assay was designed and fabricated for the purpose. Numerical modeling and micromixing experiments were performed to ascertain the mixing performance of the fabricated device. Molecular modeling was performed to demonstrate tau protein structural changes resulting from ATP binding. Tau aggregation was realized on-chip via ATP conjugation, and the MTC dose–response was studied in detail via LSCM. Drug dose optimization was performed, and the reproducibility of tau–drug experiments was verified. Finally, this work provides a microfluidic platform for detecting tau aggregation level in less than 2 h. Moreover, the device can also be upgraded to a smartphone imaged microfluidic chip, as reported by Ghonge et al. (2019) [21].

## Figures and Tables

**Table 1:** Drug concentration optimization using one factorial design.

Run	Factor 1 (Drug Dose µg/µL)	Response 1 (% of Intensity)
**1**	0.1	0.2
**2**	1.43	48
**3**	1.76	47.5
**4**	0.77	28.4
**5**	2.1	47.89
**6**	1.1	45.23
**7**	2.1	47.44
**8**	1.1	46.02
**9**	0.44	1.6
**10**	0.1	0.31

**Table 2:** Docking score representing essential parameters during ATP binding to tau.

Protein Target	Ligand	Ligand Efficiency (kcal/mol)	Inhibition Constant	Electrostatic Energy	Electrostatic Interaction	Pi-Pi Interaction
Tau	ATP	−0.02	319.75	−0.41	NA	TYR310

## Supplementary Materials

The following are available online at https://www.mdpi.com/2306-5354/7/4/162/s1, Figure S1: Microfluidic device compared with coin of radius 2.5cm as a scale; Figure S2: Spatial Intensity map for comparative study between the native tau protein and regained tau protein at drug dose 1.43 µg/mL [Representative image from the imaging window]; Figure S3: Heterogeneity in native tau protein intensity in different sections/tiles of imaging window (**a**) Tau protein intensity in each section/tile in a stitched image and (**b**) Average intensity in each stitched image, S1, S2, S3, S4 (Mean ± S.E.) for native tau protein.
