# Supplementary material for: Microfluidic Protein Imaging Platform: Study of Tau Protein Aggregation and Alzheimer’s Drug Response
Source: Bioengineering (Basel). 2020 Dec 13;7(4):162. doi: 10.3390/bioengineering7040162 (PMC7763324; doi:10.3390/bioengineering7040162)
Supplement: Supplementary file 1 [file bioengineering-07-00162-s001.pdf]

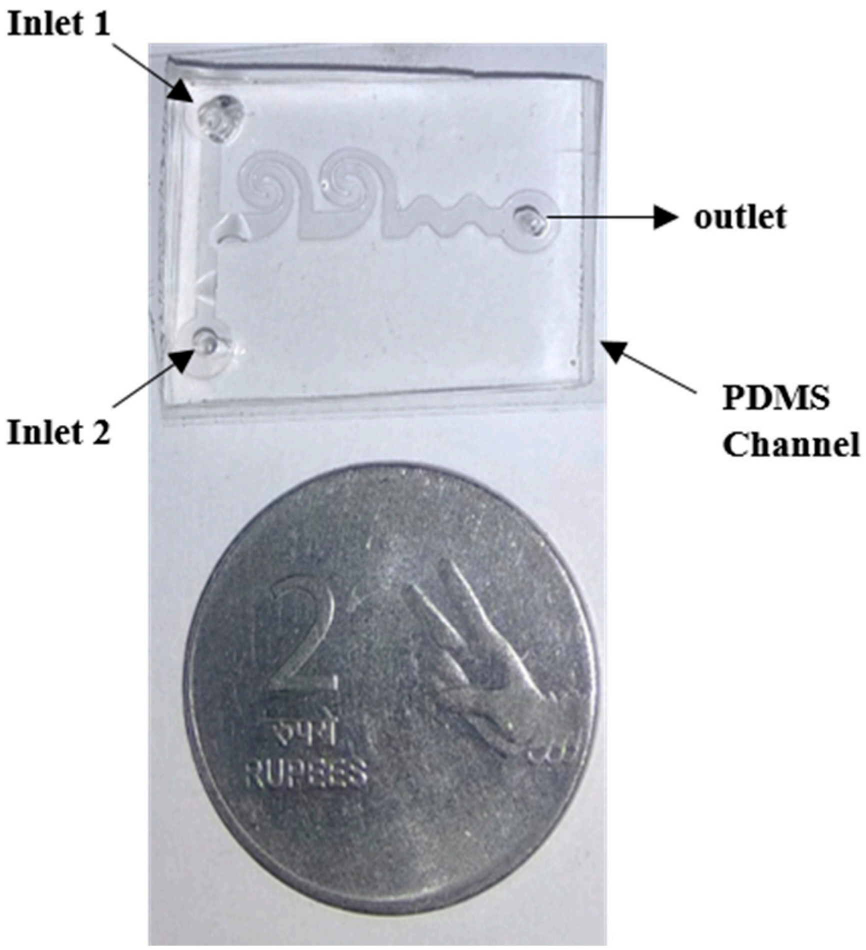

Figure S1: Microfluidic device compared with coin of radius 2.5 cm as a scale

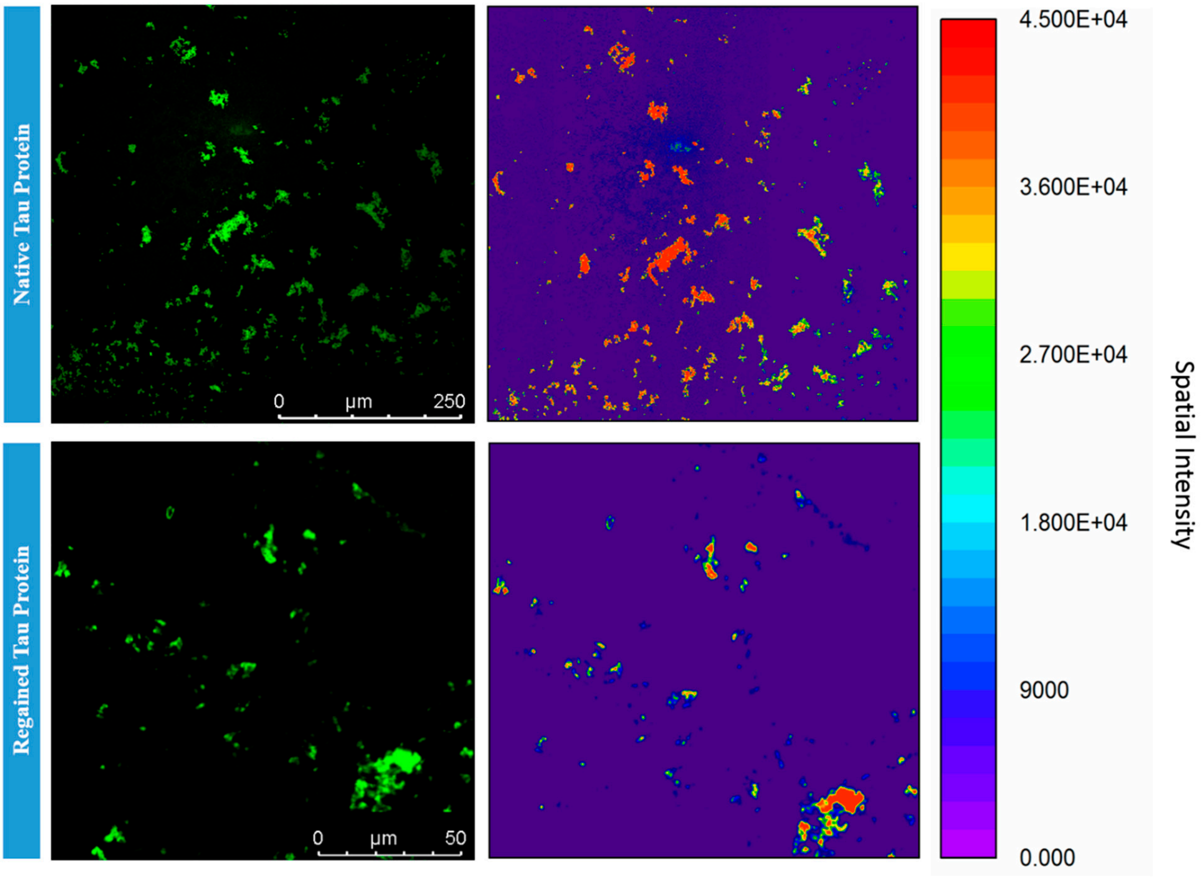

Figure S2: Spatial Intensity map for comparative study between the native tau protein and regained tau protein at drug dose 1.43 µg/µL [Representative image from the imaging window].

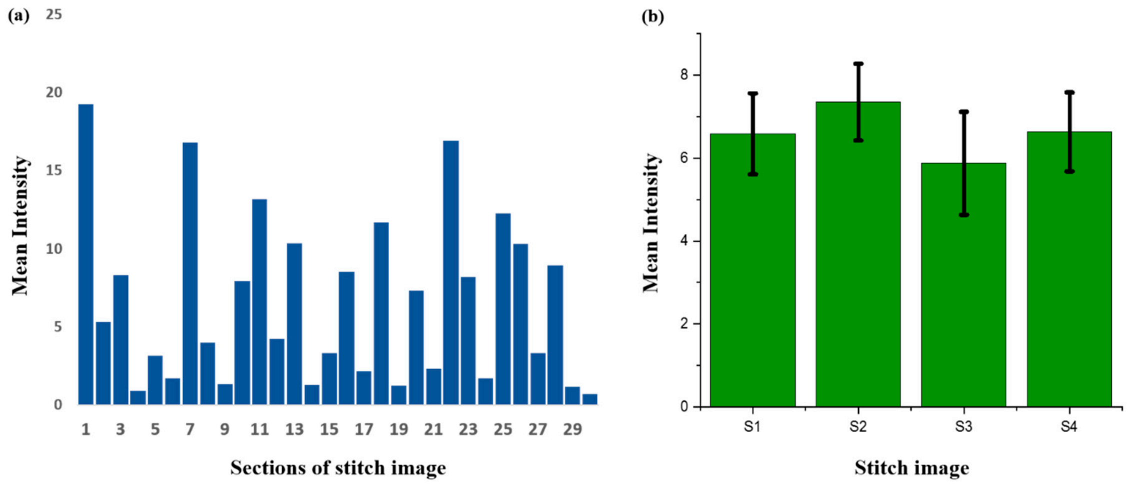

Figure S3: Heterogeneity in native tau protein intensity in different sections/tiles of imaging window (**a**) Tau protein intensity in each section/tile in a stitched image and (**b**) Average intensity in each stitched image, S1, S2, S3, S4 (Mean ± S.E.) for native tau protein.
